# Development of a Mobile Phone-Based Weight Loss Lifestyle Intervention for Filipino Americans with Type 2 Diabetes: Protocol and Early Results From the PilAm Go4Health Randomized Controlled Trial

**DOI:** 10.2196/resprot.5836

**Published:** 2016-09-08

**Authors:** Melinda Sarmiento Bender, Glenn-Milo Santos, Carissa Villanueva, Shoshana Arai

**Affiliations:** ^1^ School of Nursing Department of Family Health Care Nursing University of California San Francisco San Francisco, CA United States; ^2^ School of Nursing Department of Community Health Systems University of California San Francisco San Francisco, CA United States; ^3^ Institute for Health and Aging Department of Social and Behavioral Sciences University of California San Francisco San Francisco, CA United States

**Keywords:** randomized controlled trial, lifestyle intervention, weight loss, Filipinos, type 2 diabetes, culturally adapted, Asian Americans

## Abstract

**Background:**

Filipino Americans are the second largest Asian subgroup in the United States, and were found to have the highest prevalence of obesity and type 2 diabetes (T2D) compared to all Asian subgroups and non-Hispanic whites. In addition to genetic factors, risk factors for Filipinos that contribute to this health disparity include high sedentary rates and high fat diets. However, Filipinos are seriously underrepresented in preventive health research. Research is needed to identify effective interventions to reduce Filipino diabetes risks, subsequent comorbidities, and premature death.

**Objective:**

The overall goal of this project is to assess the feasibility and potential efficacy of the Filipino Americans Go4Health Weight Loss Program (PilAm Go4Health). This program is a culturally adapted weight loss lifestyle intervention, using digital technology for Filipinos with T2D, to reduce their risk for metabolic syndrome.

**Methods:**

This study was a 3-month mobile phone-based pilot randomized controlled trial (RCT) weight loss intervention with a wait list active control, followed by a 3-month maintenance phase design for 45 overweight Filipinos with T2D. Participants were randomized to an intervention group (n=22) or active control group (n=23), and analyses of the results are underway. The primary outcome will be percent weight change of the participants, and secondary outcomes will include changes in waist circumference, fasting plasma glucose, glycated hemoglobin A1c, physical activity, fat intake, and sugar-sweetened beverage intake. Data analyses will include descriptive statistics to describe sample characteristics and a feasibility assessment based on recruitment, adherence, and retention. Chi-square, Fisher's exact tests, t-tests, and nonparametric rank tests will be used to assess characteristics of randomized groups. Primary analyses will use analysis of covariance and linear mixed models to compare primary and secondary outcomes at 3 months, compared by arm and controlled for baseline levels.

**Results:**

Recruitment was completed in January, 2016, and participant follow-up continued through June, 2016. At baseline, mean age was 57 years, 100% (45/45) of participants self-identified as Filipinos, and the cohort was comprised of 17 males and 28 females. Overall, participants were obese with a baseline mean body mass index of 30.2 kg/m2 (standard deviation 4.9). The majority of participants were immigrants (84%, 38/45), with 47% (21/45) living in the United States for more than 10 years. One third of all participants (33%, 15/45) had previously used a pedometer.

**Conclusions:**

This study will provide preliminary evidence to determine if the PilAm Go4Health weight loss lifestyle intervention is feasible, and if the program demonstrates potential efficacy to reduce risks for metabolic syndrome in Filipinos with T2D. Positive results will lend support for a larger RCT to evaluate the effectiveness of the PilAm Go4Health intervention for Filipinos.

**ClinicalTrial:**

ClinicalTrials.gov: NCT02290184; https://clinicaltrials.gov/ct2/show/NCT02290184 (Archived at http://www.webcitation.org/6k1kUqKSP)

## Introduction

### Objectives

The Filipino Americans Go4Health Weight Loss Program (PilAm Go4Health) study is a 3-month pilot randomized controlled trial (RCT), evaluating a weight loss lifestyle intervention, with a 3-month maintenance phase and active waitlist control. This program is a mobile phone-based intervention that includes virtual social support, promotes physical activity (PA) and healthy eating, and was culturally adapted specifically for Filipino Americans. The overall objective of this trial is to assess the feasibility and potential efficacy of the PilAm Go4Health intervention to reduce risks for metabolic syndrome in overweight Filipinos with non-insulin dependent type 2 diabetes (T2D). Metabolic syndrome is a cluster of physical conditions (eg, obesity, hypertension, diabetes) that together increase the incidence of chronic diseases such as stroke and cardiovascular disease [1].

The purpose of this paper is to describe the design, cultural adaptation, and procedures of the PilAm Go4Health intervention. In addition, participant baseline characteristics, lessons learned from community stakeholder input, and recruitment strategies will be provided. This information will inform the development of future culturally relevant lifestyle interventions, and improve participant engagement and retention among hard to reach populations (particularly Filipinos).

### Background

Filipinos are the second largest US Asian subgroup, totaling 3.4 million people [2]. This population suffers from some of the highest prevalences of obesity, T2D, and cardiovascular disease compared to most Asian American subgroups and non-Hispanic Whites [3,4]. Major contributors to the high prevalence of Filipino obesity-related chronic diseases include a genetic predisposition to abdominal fat distribution, cultural preferences for a high fat diet, and sedentary behavior [5,6]. Furthermore, as a community-oriented society, Filipinos harbor cultural beliefs with insular tendencies that limit their willingness to readily engage in Western health care practices [7,8]. Although Filipinos are one of the fastest growing US immigrant racial/ethnic populations, they are seriously underrepresented in preventive health research [9]. Given the high prevalence of chronic disease in this high-risk population and the escalating costs of health care, it is imperative to identify effective intervention strategies to reduce these preventable health disparities.

Risk-reduction interventions such as the Diabetes Prevention Program (DPP) have been effective in delaying and reducing diabetes risks, even with modest amounts of weight loss and PA [10]. Recent systematic reviews have revealed that digital technologies using mobile phone-based interventions have the potential to promote both PA and weight loss [11,12]. Furthermore, a recent Cochrane review reported that mobile phone-based interventions had a beneficial effect on blood glucose control in self-management of T2D [13]. Currently there are over 97,000 commercial electronic mobile health (mHealth) apps available to the public [14]. To date, only a limited number of studies have evaluated the effectiveness of these commercial mHealth apps with regards to the promotion of healthy lifestyle behaviors.

Despite the success of the DPP in reducing diabetes risks, DPP guidelines require frequent face-to-face visits (16 or more) that are labor-intensive and burdensome to health care staff and patients alike [15]. Leveraging digital technology with lifestyle interventions is an ideal strategy to address the issue of frequent labor-intensive face-to-face visits, particularly among Filipinos, given their propensity to use smartphones and virtual social media (eg, Facebook) [16]. Remote education and coaching, along with virtual social support, would require fewer in-person meetings. A recent comparison of digital technology usage among Filipinos, Hispanics, Koreans, and Whites found that Filipinos were consistently ranked first or second as the most prolific users of smartphones, iPads/tablets, email, mobile apps, and social media platforms (eg, Facebook) [17]. In light of the existing health disparities and dearth of effective preventive health strategies for Filipinos, the prolific use of digital technology among Filipinos makes them ideal candidates for mHealth-supported lifestyle interventions to help mitigate their diabetes prevalence and cardio-metabolic risks [16]. Therefore, we adapted the original DPP (while retaining its core fundamentals) and created a culturally relevant mobile phone-based weight loss lifestyle intervention that includes virtual social networking for Filipinos. By leveraging advances in mobile technology, along with the rapid penetration of smartphone use among Filipinos and their propensity for Facebook social networking, we reduced the required number of DPP in-person meetings, resulting in a less labor-intensive intervention with the potential to improve cost-effectiveness.

### Research Benefits and Impact

The PilAm Go4Helath study offers three important benefits to science. First, to our knowledge the PilAm Go4Health weight loss trial is the first culturally adapted mobile phone-based lifestyle intervention using digital technology that focusses on Filipinos’ virtual and in-person social networks to support target health behaviors. This multi-pronged social support intervention integrates in-person meetings, family participation, and a private Facebook group designed exclusively for study participants. The private Facebook group coalesces the following support elements: text and photos for education and coaching; real-time monitoring to promote target health behaviors; and weekly positive feedback and motivational messages to improve participant adherence and retention.

Second, trial findings will report on the efficacy of the PilAm Go4Health lifestyle intervention, which incorporates the commercially available Fitbit Zip (and corresponding mHealth app) to promote weight loss through PA and healthy eating. The Fitbit Zip is a state-of-the-art lightweight accelerometer/altimeter that is paired with an associated mHealth app to record and wirelessly transmit real-time step-count data. Use of the commercial Fitbit Zip will facilitate dissemination of our findings to a broader consumer population of Fitbit users.

Third, a community engagement approach was used to assist in the development of a culturally relevant intervention to enhance acceptability and improve participant recruitment, engagement, and retention [18,19]. Findings from the process that was used to culturally adapt the PilAm Go4Health intervention for Filipinos, and other strategies that were used for recruitment, engagement, and retention, may be useful for other research investigators and health workers when planning and developing health-related interventions for other racial/ethnic and diverse populations.

## Methods

### Study Design

**Figure 1:**
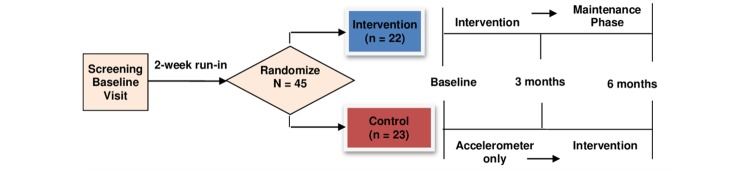
PilAm Go4Health Study design.

This study was a 3-month pilot RCT with an active waitlist control and a 3-month maintenance design (Figure 1) **.** The objective of this trial is to assess the feasibility and potential efficacy of the PilAm Go4Health intervention to reduce risks for metabolic syndrome in Filipinos with T2D. The primary aims are to: (1) assess the feasibility of a mobile phone-based lifestyle intervention using digital technology (accelerometer, mHealth app, and private Facebook group) as measured by participant recruitment, engagement, adherence, and retention; (2) determine preliminary estimates of the effect of primary outcomes (percent weight) and secondary outcomes (waist circumference, fasting plasma glucose, glycated hemoglobin A1c [HbA1c] levels, PA, and diet [reduced fat and sugar-sweetened beverage intake]); and (3) conduct post-program process evaluations to obtain participant feedback regarding cultural relevancy, barriers to adherence, perceived efficacy for healthy behaviors, and suggestions for intervention improvements.

Cultural adaptations to the PilAm Go4Health study design were based on stakeholder feedback. During discussions with community leaders and members prior to study implementation, we learned that Filipinos are less likely to participate in research studies if they are randomized to a control group that does not receive the intervention. Therefore, to incentivize participant recruitment, engagement, and retention, the control group was waitlisted to receive the PilAm Go4Health intervention immediately after completing the 3-month control period.

Prior to implementation, the study protocol was reviewed and approved as a minimal risk study by the University of California San Francisco Institutional Review Board/Committee on Human Subjects Research.

### Conceptual Framework

Social cognitive theory was a guide for the PilAm Go4Health study design [20]. This theory posits that behavior change is influenced by one’s environment (eg, socio-cultural and community). Individual demographics, acculturation, and health literacy factors may also influence behaviors, while environmental feedback (social support, whether positive or negative) can reinforce or discourage behaviors. Social support may also affect self-efficacy for a given behavior; self-efficacy is the confidence to successfully reproduce target behaviors (eg, increase PA and reduce high fat/calorie intake for weight loss). PilAm Go4Heatlh uses social networking (virtual and in-person meetings with family and peers) to promote self-efficacy for healthy behaviors and weight loss.

### Sample

The proposed sample size of 45 is conventional for pilot studies, with attrition estimated at 10%, for a final sample of 40 participants. Inclusion and exclusion criteria were based on American Heart Association metabolic syndrome risks, diagnosis, and management, as well as the DPP trial [1,10]. Key inclusion criteria included: (1) self-identified as Filipino; (2) age >18 years; (3) World Health Organization body mass index (BMI) cut-point for Asians >23 kg/m^2^for public health action [21]; (4) physician diagnosed T2D confirmed by clinical data (eg, documentation of fasting blood glucose >100 mg/dL, a positive oral glucose tolerance test >200 mg/dL, or HbA1c >6.5%); (5) noninsulin dependent T2D; and (6) own a smartphone, tablet, or laptop computer. Key exclusion criteria included: (1) uncontrolled T2D (fasting blood glucose >200 mg/dL); (2) disabilities precluding ability to walk for 20 minutes; (3) glucose metabolism-associated disease (Cushing's syndrome, Acromegaly, or Pheochromocytoma currently under treatment, or chronic pancreatitis). A detailed list of inclusion/exclusion criteria are presented in Multimedia Appendix 1.

### Recruitment

Study recruitment began in December, 2014, and all baseline assessments were completed in January, 2016. Participants were recruited from San Francisco and Daly City's Filipino communities. Filipinos represent 4.6% of San Francisco’s population (32,268/806,696) and 34.5% of Daly City’s population (34,998/101,443), the latter being the largest concentration of Filipinos in the United States [22,23].

### Primary Recruitment Plan

Key aspects of the primary recruitment plan included: (1) contacting pre-identified potential Filipino participants (over 250 respondents) from the San Francisco Bay Area who expressed interest while participating in a prior study [17]; (2) offering diabetes education classes at Filipino community centers (eg, Lions clubs and faith-based organizations); (3) distributing study fliers at Filipino community events; (4) posting study flyers on public websites (eg, Craigslist, Filipino organization websites); and (5) advertising at Filipino community centers, faith-based organizations, ethnic markets, libraries, laundromats, community colleges and universities, and at local health care facilities (clinics and hospitals).

### Alternate Recruitment Plan

The alternate recruitment plan included: (1) sending study invitations to potential participants receiving services at medical centers, primary care clinics, and diabetes clinics; (2) using a commercial mailing service to send study invitations to Filipino residents living in select zip codes listed in the publicly available US census database; and (3) distributing flyers at Daly City Chamber of Commerce events.

### Intervention Strategies

This intervention incorporated the following four diabetes prevention strategies: (1) weight loss through PA and diet; (2) cultural tailoring of the intervention; (3) leveraging digital technology; and (4) integrating social support.

#### Weight Loss Through Physical Activity and Diet

This study was a 6-month intervention (3-month weight loss program with a 3-month maintenance period) to reduce weight and thereby diabetes risks via increasing PA (weekly step-count goals) and diet (lowering fat and sugary-beverage intake). Participants’ goals were individually tailored from their baseline weight, weekly step-counts, weekly sugary beverage intake, and daily fat intake. Individual goals were to lose 5% body weight from baseline, increase and maintain steps up to 12,000 steps/day (increase weekly step-count goal by 20% based on total step-counts from the previous week), reduce sugary beverage intake (to once/week or less), and reduce total daily fat intake (to 25% of total calories from fat/day). The 3-month maintenance stage aimed to ensure long-term adherence to target health behaviors and maintain weight loss.

#### Cultural Tailoring of the Intervention

Cultural relevance of an intervention enhances effectiveness and acceptability for the target population by improving participant recruitment, engagement, and retention [18]. Cultural adaptations to the intervention were made, based on previously published data: Bender et al published guidelines in 2011 [19] and a recent 2015 study profiling digital technology use among diverse populations [17]; the National Heart, Lung, and Blood Institute (NHLBI) recommendations for culturally adapting Filipino American interventions [24]; and the NHLBI Filipino education materials for reducing risks of cardiovascular disease [25].

Additional tailoring, based on stakeholder (community members, leaders, and health care providers) feedback, incorporated relevant Filipino language, food, PA options, and social support. These factors included: social support in the form of in-person meetings that incorporated family members and virtual social support (private Facebook group); common Filipino PAs (ie, walking, dancing, and/or basketball); and healthy Filipino food alternatives such as roasted chicken, grilled fish, and brown rice (to replace fried pork, sausage, and white rice, respectively). To improve awareness and assist in tracking total daily calories consumed, a photo booklet was developed that included pictures of common Filipino foods, dishes, and beverages, along with the total calories and fat content for each item.

Materials were provided in English, the second official national language of the Philippines. English is taught in all Philippine schools and over 90% of Filipinos are proficient in English [7,26]. To deliver the intervention, the research team included members of Filipino descent who were trusted members of the community and were familiar with cultural values, community norms, and Tagalog language.

#### Leveraging Digital Technology: Fitbit Zip Accelerometer and mHealth App/Diary

Direct-to-consumer mHealth wearable devices pose multiple benefits for behavioral research. Self-monitoring and tracking of lifestyle behaviors (eg, PA and weight) have been shown to improve weight loss and health outcomes [12,27]. Therefore, the Fitbit Zip mHealth self-monitoring lifestyle behavior tracker with an associated mHealth app was chosen, based on recommendations from previous studies [28-31]. These studies demonstrated that the Fitbit encouraged high levels of adherence to self-monitoring of PA, exhibited high inter-device reliability for tracking PA step-counts, and was acceptable among participants. Furthermore, the Fitbit Zip is commercially available, affordable, and scalable.

The Fitbit Zip is a state-of-the-art lightweight accelerometer/altimeter sensor that wirelessly transmits real-time data for PA (step-counts, distance, duration, and energy expenditure in Metabolic Equivalent of Task [1 kcal/kg/hour]) and sleep (not measured in this study). An associated mHealth Fitbit app/diary is available for participants to input and self-report their daily food/drink type and calorie intake, and weekly weight. Real time feedback is provided by the Fitbit app in the form of chart and graph displays of lifestyle behaviors (eg, step-counts and calories consumed). These capabilities and features may help enhance participant self-report quality, prevent recall bias, and promote adherence and retention.

#### Integrating Social Support - Two Modalities

Filipinos place a premium on community, family, and social support [7]. Moreover, family and peer support are known to improve healthy behavior motivation, adherence, and maintenance [32,33]. Thus, to enhance social support, PilAm Go4Health included two social modalities: (1) in-person intervention sessions welcoming family participation, and (2) a virtual Facebook social networking group.

##### Mode 1: In-Person Intervention

This modality consisted of four individual office visits. The first visit (individual baseline randomization) focused on tailored short-term and long-term goals for the subject. To enhance social support for target behaviors, study subjects were asked to attend additional office meetings once per month for three months (Table 1). To lend support and encouragement, the intervention group’s family members were welcomed to attend the in-person office visits. Family attendance at meetings was recorded.

**Table 1:** Intervention modalities.

Social Support Modality	Time Frame	Parameters
**Mode 1: In-Person Intervention Sessions**		
	Baseline Randomization (Individual)	Lifestyle balance and social networking
		Initiating physical activity and healthy diet
		Setting short-term and long-term goals
	1 Month (Group)	Benefits and ways to be physically active
		Social support for physical activity
		Culturally relevant physical activities
	2 Month (Group)	Benefits and ways to eat healthy
		Limiting fat intake and healthy eating out
		Healthy Filipino food alternatives
	3 Month (Group)	Relapse prevention
		Problem solving and staying motivated
		Social support for healthy behaviors
**Mode 2: Virtual Social Networking Group**		
	Ongoing Facebook chat room, baseline to 3-month (Group)	Administered and monitored by staff
		Only intervention participants invited
		12 weekly posted discussion topics
		Post educational materials
		Participants can share messages and photos

##### Mode 2: Virtual Private Facebook Group

**Figure 2:**
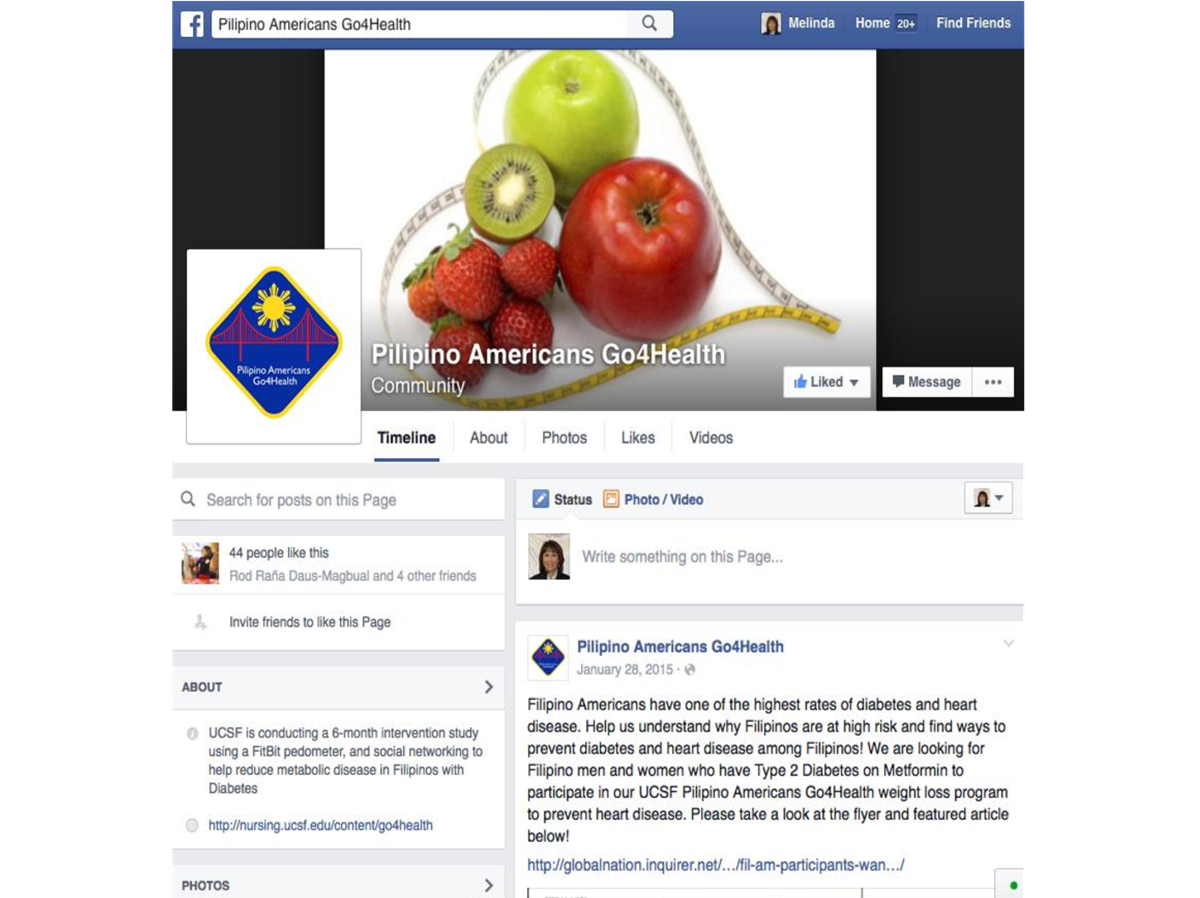
PilAm Go4Health Facebook Page.

Facebook is an online social network service with multiple interactive capabilities. To ensure privacy and maintain Health Insurance Portability and Accountability Act compliance, a PilAm Go4Health private Facebook group was created. This private Facebook group could be accessed through smartphones, tablets, or laptops, and was available only to PilAm Go4Health intervention subjects. The Facebook group was designed to encourage group interactions, bonding, and social support to help subjects achieve their short-term and long-term program goals, overcome barriers, and prevent relapse. Research staff administered, monitored, and moderated the PilAm Go4Health Facebook group. Each member’s usage (number and frequency of logins, likes, and posts) was monitored and research staff posted weekly topics along with photos that promoted target health behaviors, and facilitated and oversaw ongoing discussions. Facebook subjects received a Facebook notification whenever a message was posted on the site. *Weekly topics* were adapted from the DPP core curriculum and the NHLBI Health Heart Education for Filipino Communities related to PA and diet [15,25]. Subjects were encouraged to post healthy Filipino recipes and photos. Topics included reducing fat with healthy Filipino food alternatives, and benefits of tracking step-counts, calories, food/drinks, and weight on the Fitbit app/diary. See Figure 2 for a PilAm Go4Health Facebook screenshot.

### Intervention Protocol

Intervention and control group subjects received a total of seven in-person office visits (Figure 1). After the screening baseline visit (SBV) and randomization visit, the intervention group received three intervention follow-up visits, and two maintenance follow-up visits. The control group received one control follow-up visit and three follow-up visits after the 3-month transition, in order to receive the intervention program.

#### Screening Baseline Visit

Potential subjects were screened by phone interview after providing verbal consent. Those who met all inclusion criteria were scheduled for an SBV. After administering informed consent, subjects attending the SBV were asked to answer questionnaires and provide sociodemographic data **,** consent to a brief physical exam and a fasting blood draw for study measures, receive a Fitbit Zip accelerometer, and attend a mobile phone and Fitbit Zip training session.

#### Run-In Period

After the SBV, eligible subjects immediately started a two-week run-in period, during which they were asked to wear a Fitbit Zip every day for at least 10 hours/day, and send photos of all food and drinks consumed for three consecutive days. Study eligibility required a 70% adherence rate, at minimum.

#### Fitbit Zip and App/Diary Training

Prior to starting the run-in period, eligible subjects received training on how to use the Fitbit Zip and download the app. Subjects who did not own a smartphone used their own tablets or personal laptops. At the randomization visit, only intervention group subjects were trained on how to download and use the Fitbit app/diary, and asked to input their daily food/drinks and calorie intake, and weekly weight. Active control group subjects were only trained to use the Fitbit Zip and download the app until the 3-month time point. After the 3-month control period, control subjects were trained to use the Fitbit app/diary when they received the intervention program.

All participants were asked to provide an overnight plan to store their Fitbit Zip. This information was used to initiate a recovery protocol if equipment was lost, and included a comprehensive interview to help subjects reconstruct step-by-step events of the previous 24 hours. Based on previous studies, this recovery protocol was 98% successful [29,30].

#### Facebook Training

Subjects receiving the intervention program received training to access and use the private Facebook group, with education on appropriate Facebook etiquette. Prior to joining the private Facebook group, each subject was assigned a secure email account (excluding personal information) for the PilAm Go4Health private Facebook group.

#### Randomization and Blinding

At the end of the two-week run-in period, subjects able to adhere to the run-in requirements 70% of the time were randomized to either the intervention group or active control group in a 1:1 ratio. A permuted block randomization method stratified by gender was used with randomly selected block sizes of 2 and 4. Prior to the trial, the study statistician created a computer-generated random allocation sequence. Investigators, research staff (who conducted anthropometric measures, entered data, and undertook data management), and study participants were unblinded during the study, but personnel performing the lab sample evaluations were blinded to treatment groups.

##### Intervention Group

Only subjects randomized to the intervention group immediately received the PilAm Go4Health intervention. Participants were asked to wear their Fitbit Zip at least 10 hours/day and use the Fitbit app/diary to input and track their daily food/drink and calorie intake, and to record their weight twice per week (Monday and Friday). Subjects received individually tailored goals, in-person intervention meetings, Fitbit Zip and Fitbit app/dairy training, and private Facebook group training (and an invitation to join the study Facebook group). Each subject’s baseline weight, PA, and diet information were used to tailor their intervention for short-term and long-term goals. Based on each subject’s progress during the study, they received tailored feedback and coaching, including barrier and support assessments, and information regarding the benefits of tracking health behaviors on the Fitbit Zip and Fitbit app/diary. Research staff monitored each subject’s Fitbit Zip and Fitbit app/diary data (step-counts, food/drink and calorie intake, and weight), and Facebook logins, likes, and posts. All automatically recorded Fitbit Zip data were wirelessly uploaded to each subject’s individual study Fitbit and Facebook account for storage on study data servers. Table 1 displays an overview of topics covered at each in-person office visit, and during private Facebook group participation.

##### Active Control Group

Subjects randomized to the active control group were asked to continue to only use the Fitbit Zip every day. During the randomization visit, each active control participant received Hepatitis B education, including the National Digestive Diseases Information - Hepatitis B handout for Asians and Pacific Islanders [34].

#### Office Visits - 1 Month Through 6 Months

After the randomization visit, intervention group subjects were asked to return once per month for three months, for intervention education, coaching, and support. At the 3-month visit, subjects transitioned to the 3-month maintenance, and were asked to return for a 4-month progress evaluation and 6-month office visit. Active control group subjects were asked to return at the 1-month visit for a progress evaluation, and received Hepatitis C education. After completing the 3-month control period, active control subjects transitioned to receive the PilAm Go4Health intervention, and were asked to return for intervention follow-up visits at four, five, and six months. At the final 6-month office visit, all subjects completed the PilAm Go4Health study (see Figure 1).

At the 3-month office visit, all intervention and control subjects were asked to answer questionnaires and consent to a physical exam and fasting blood draw. Intervention subjects were asked to participate in a post-program process evaluation (semi-structured interview) before transitioning to the 3-month maintenance program.

At the 6-month office visit, all intervention and control group subjects were asked to complete questionnaires, consent to a physical exam and a final fasting blood draw, and participate in a post-program process evaluation (semi-structured interview). All subjects completed the study at this visit, and were removed from the Fitbit study account and private Facebook group account. Subjects who completed the 6-month study were allowed to keep their Fitbit Zip, and research staff assisted subjects in setting up their own personal Fitbit accounts.

### Outcome Measures

Primary and secondary outcomes were measured at baseline, 3 months, and 6 months. Process evaluations were collected only at 3 and 6 months. During the analysis phase of this project, the primary outcome will be percent change in body weight, while secondary outcomes include changes in waist circumference, fasting plasma glucose, HbA1c levels, step counts, and dietary fat and sugar-sweetened beverage intake. Participants also provided feedback on the intervention program regarding cultural relevancy, compliance to the mobile phone-based intervention, compliance to the virtual social networking, barriers to use, perceived efficacy, and suggestions for intervention improvements.

In addition to the primary and secondary outcomes, self-reported information regarding self-efficacy for PA and social support were collected at baseline, 3 months, and 6 months. Sociodemographics, health literacy, and acculturation data were collected only at baseline. Table 2 presents all study questionnaires, planned administration, evidence of validity and reliability, and references.

**Table 2:** PilAm Go4Health outcome measures and surveys/questionnaires.

Outcomes		Time of collection	Scale / Collection Method
**Primary Outcomes**			
	BMI, weight, height, and waist circumference	Baseline, 3 months, and 6 months	(1) Weight measured with Professional Digital Floor Scale
			(2) Height measured with Healthometer PORTROD Height Rod
			(3) Waist circumference (standing) measured with a tape measure midway between lower rib margin and iliac crest [35]
			(4) BMI = kg/m2
	Fasting plasma glucose & HbA1c	Baseline, 3 months, and 6 months	Venipuncture blood specimen collected at the Clinical and Translational Science Institute Clinical Research Service Center
**Secondary Outcomes**			
	Physical activity	Baseline, 3 months, and 6 months	(1) Objectively measured total daily step-counts collected by the Fitbit Zip and mHealth app
			(2) Self-reported International Physical Activity Questionnaire [36] - 27 items (Cronbach alpha median 0.80)
	Diet	Baseline, 3 months, and 6 months	(1) Fat-Related Diet Habits Questionnaire [35,37] - 22 items (Cronbach’s alpha = 0.77)
			(2) Beverage Intake Questionnaire [38] - 15 items (Cronbach’s alpha range 0.97 to 0.99)
**Other Outcomes**			
	Self-efficacy for physical activity	Baseline, 3 months, and 6 months	Self-efficacy for Physical Activity Questionnaire [39] - 12 items (Cronbach alpha range 0.78 to 0.82)
	Self-efficacy for diet	Baseline, 3 months, and 6 months	Eating Confidence survey [39,40] -16 items (Cronbach alpha range 0.83 to 0.84)
	Social support for diet and exercise	Baseline, 3 months, and 6 months	Social Support for Diet and Exercise Survey [41] – 23 items (Cronbach alpha range 0.80 to 0.93)
	Self-reported health	Baseline, 3 months, and 6 months	Patient-Reported Outcomes Measurement Information System Survey Global Health [42] - 10 items (internal consistency=0.81, validity=0.86)
	Process evaluations	3 months and 6 months	Comprehensive, semi-structured interviews to solicit participant feedback on cultural relevancy, compliance to the mobile intervention, barriers to use, and suggestions for intervention improvements (at 3 and 6 months)
**Baseline Outcomes**			
	Sociodemographics and medical history	Baseline only	Gender, ethnicity, birth date, primary language, years lived in United States, marital status, number of children, education level, employment and health insurance status, income, hyperlipidemia, smoking, hypertension or other chronic illnesses, and/or family history of diabetes
	Acculturation	Baseline only	Marin Short-Acculturation Scale for Filipino Americans [43] - 12 items (Cronbach alpha range 0.79 to 0.85)
	Health literacy	Baseline only	Short-Health Literacy Survey [44,45] - 3 items (Reliability range 0.74 to 0.84)

### Data Collection

Data were collected during in-person office visits, via self-reported PA and diet, and through the Fitbit Zip and Fitbit mHealth app/diary. In-person data included questionnaires and anthropometric data that were collected during research office visits. Self-reported PA and diet included questionnaires on PA (days per week, type, and duration of exercise), and diet (sugar-sweetened beverage intake, fat related diet intake) that will be collected along with self-efficacy and social support for phsycial activity. See Table 2 for questionnaire details. All study data were entered via encrypted computers and transferred to the research study's secure database servers. Likewise, participants’ oral responses obtained from semi-structured interviews will be coded and stored on the same secure encrypted research study database servers. Fitbit Zip and app/diary data were wirelessly uploaded and transmitted in real-time directly to secure Fitbit database servers that identify participants only by their assigned Gmail account (no personal information was transferred). In turn, the study Fitbit account was tied to a secondary Fitabase database server that included all participants in our secure study Fitbit account. Fitabase is a secure confidential research platform that collects data from Internet-connected consumer devices, such as Fitbit accelerometers and mHealth apps. Fitabase aggregates data, allowing researchers to easily organize, analyze, and export data gathered from study participants. Research staff have access to all participants’ Fitbit data (step-counts, weights, and food/calorie intake) through the study Fitabase account.

### Data Analyses

Regarding aim 1 of the study, feasibility will be assessed based on recruitment (eligible participants who were screened, completed the run-in, enrolled, and randomized), adherence (during the 3-month intervention, at least 70% compliance with the Fitbit Zip, mHealth app, and weight and calorie diary), and retention (6-month program retention and attrition rate). All results will be tabulated by study arm, and reported with descriptive statistics and effect sizes or 95% confidence intervals.

Descriptive statistics will be used to analyze the sample characteristics pertaining to aim 2 of the study. Prior to the analyses, validity of the randomization will be checked by assessing the randomized groups for characteristics measured before randomization using chi-square, Fisher's exact tests, t-tests, and nonparametric rank tests, as appropriate. If potentially confounding imbalances are found, we will adjust the between-group analyses for potential confounders of treatment assignment. The primary analysis will use analysis of covariance to compare primary and secondary outcomes at 3 months by arm, controlling for baseline levels. Outcomes will be transformed as necessary to meet assumptions of normality and equal variance. In a supplementary analysis (for more efficient data use), we will include on-treatment outcomes analysis for the control group at 6 months, using a linear mixed model to account for the repeated measures on waitlist controls, and include a smooth function of time to reduce potential bias from secular effects. In addition, we will assess within-group changes in the immediate intervention group between 3 and 6 months, as a preliminary measure of the durability of the intervention effect in the maintenance phase. We will also use these models to estimate residual variances and within-person correlation, which will be crucial to future trial planning. Exploratory mediation analysis will be conducted to test the association between step-counts and diet on change in outcomes.

Aim 3 will be assessed using semi-structured interview methods. At the 3-month post-intervention time point, participants’ qualitative perspectives on their experience during the intervention (advantages, barriers, and support) and use of mobile technology (perceived usefulness, ease of use) were obtained. Qualitative data will be coded and analyzed. Transcripts will be independently coded for initial themes and then checked for inter-rater agreement by at least two research investigators. Content analysis will be conducted by marking and categorizing key words and phrases to identify emerging themes [46,47]. Data analysis will identify links across themes. Themes will be reviewed, discussed, and resolved through consensus. The research team will meet to review and discuss transcript-coding, achievement of data saturation, consensus regarding identification and definition of themes, and selection of illustrative excerpts from transcripts. Dependability of the data interpretation will be supported by investigator triangulation, a process whereby more than one investigator analyzes the data [48]. Themes derived from the data will be used to refine and further culturally tailor the intervention, in preparation of a larger full-scale RCT proposal.

## Results

### Baseline Data

**Figure 3:**
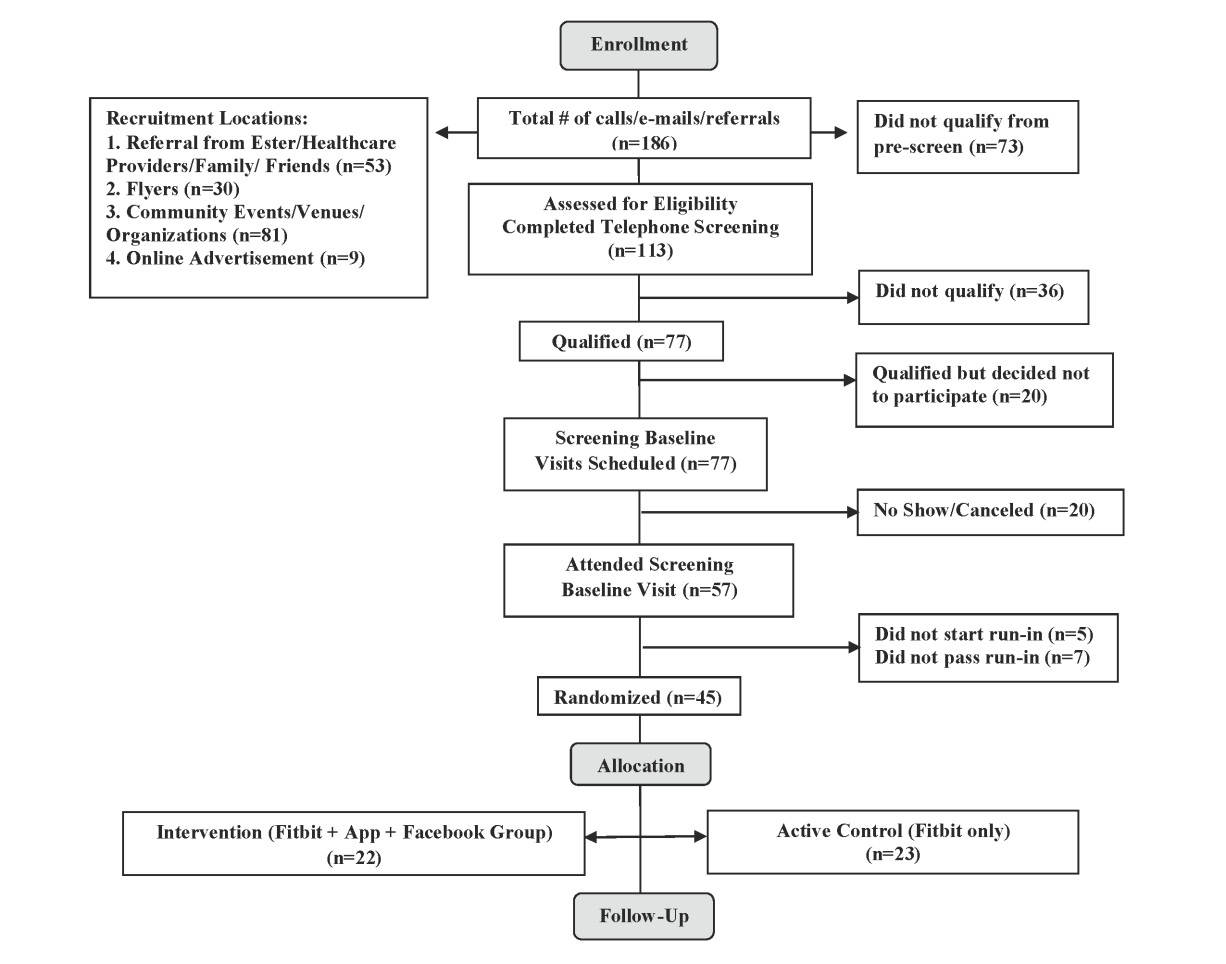
Consort Flow Diagram.

Study participants were recruited, enrolled, and randomized between December, 2014 and January, 2016. Of the 57 potentially eligible participants who completed the SBV and two-week run-in period, 45 were eligible for study enrollment, and were randomized into one of two groups: the PilAm Go4Health intervention group (n=22) or the active control group (n=23). See Figure 3 for details.

Table 3 presents the baseline characteristics of study participants. Overall, the mean age of participants was 57.6 (standard deviation [SD] 9.8) years with approximately one-third of the cohort being male (38%, 17/45) and two-thirds female (62%, 28/45). More than half of all participants had completed college (56%, 25/45), were married/cohabitating (67%, 30/45), and employed full or part time (69%, 31/45). The majority of participants were Filipino immigrants (84%, 38/45) who had lived in the United States for more than 10 years, and close to half (47%, 21/45) had 3-to-5 people living in their household. Overall, participants were in the obese BMI category, with a mean BMI of 30.2 kg/m^2^(SD 4.9) and were in the high disease-risk category with a mean waist circumference of 100.0 cm (SD 11.5) [49]. The mean fasting blood sugar level was 140.4 mg/dL (SD 27.5) and mean HbA1c was 7.4 (SD 0.99) within blood test levels for diabetes [50]. Participants in the intervention group were all Filipino immigrants (100%, 22/22), whereas 30% (7/23) of the active control group participants were born in the United States. Comparisons of baseline characteristics demonstrated two statistically significant differences (*P*<.05) between the intervention and control groups: years lived in the United States, and BMI.

**Table 3:** Baseline sociodemographics, anthropometrics, and serum lab results.

Variable		N=45 Mean (SD) or % (n)	Intervention N=22 Mean (SD) or % (n)	Control N=23 Mean (SD) or % (n)	*P*-value
Age in years		57.6 (9.8)	57.4 (9.8)	57.7 (10.0)	0.90
Race (Filipino)		100 (45)	100 (22)	100 (23)	1
Gender					0.85
	Male	37.8 (17)	36.4 (8)	39.1 (9)	
	Female	62.2 (28)	63.6 (14)	60.9 (14)	
Marital status					0.063
	Never married	11.1 (5)	4.5 (1)	17.4 (4)	
	Divorced/widowed	22.2 (10)	31.8 (7)	13.0 (3)	
	Married/cohabitating	66.7 (30)	63.6 (14)	69.6 (16)	
Education					0.67
	Some college	24.4 (11)	18.2 (4)	30.4 (7)	
	Completed college	55.6 (25)	63.6 (14)	47.8 (11)	
	Graduate school	20.0 (9)	18.2 (4)	21.7 (5)	
Employed					0.21
	Full or part time	68.9 (31)	77.3 (17)	60.9 (14)	
	Unemployed	4.4 (2)	4.5 (1)	4.3 (1)	
	Retired	26.7 (12)	18.2 (4)	34.8 (8)	
Years lived in the United States					0.003*
	Native born	15.6 (7)	0 (0)	30.4 (7)	
	5 to 9 years	2.2 (1)	0 (0)	4.3 (1)	
	10 years or more	82.2 (37)	100 (22)	65.2 (15)	
Number living at home					0.82
	<3	42.3 (19)	40.9 (9)	43.5 (10)	
	3 to 5	46.7 (21)	50.0 (11)	43.5 (10)	
	>5	11.1 (5)	9.1 (2)	13.0 (3)	
Previous pedometer use					0.41
	Yes	33.3 (15)	27.3 (6)	39.1 (9)	
	No	66.7 (30)	72.7 (16)	60.9 (14)	
Outcome measures					
	Weight (kg)	75.8 (17.0)	71.6 (9.1)	80.3 (22.3)	0.21
	Waist circumference (cm)	100.0 (11.5)	97.4 (8.2)	102.8 (14.1)	0.24
	BMI (kg/m^2^)	30.2 (4.9)	28.4 (3.3)	32.2 (5.7)	0.048*
	Fasting glucose (mg/dl)	140.4 (27.5)	135.8 (23.1)	145.3 (31.7)	0.39
	HbA1c (%)	7.40 (0.99)	7.26 (0.89)	7.55 (1.09)	0.45
	Steps (steps/day)	7431 (1789)	7142 (1475)	7743 (2091)	0.40

* *P*<0.05 between group difference in baseline characteristics.

## Discussion

The objective of this 3-month pilot RCT, with a wait-list control and 3-month maintenance phase, was to assess the feasibility and potential efficacy of the PilAm Go4Health program to reduce risks for metabolic syndrome in Filipinos with T2D. To our knowledge, this is the first such culturally adapted intervention program incorporating digital technology specifically targeting Filipinos with T2D that are at high risk for metabolic syndrome. Data collected thus far demonstrates feasibility for recruiting this specific, hard to reach population to participate in a weight loss program that promotes PA and healthy eating. Participant referral, primarily through word of mouth from family and community members, and face-to-face recruitment through community cultural events, were the most effective approaches, indicating a high level of interpersonal contact that promotes trust may have been needed to motivate study participation.

### Lessons Learned with Recruitment Strategies

Overall, recruitment was challenging, requiring approximately one year for this hard to reach Filipino population with T2D. One problematic barrier was due to the study's rigorous inclusion/exclusion criteria (see Multimedia Appendix 1) that precluded those with frequently occurring T2D comorbidities such as gout, hypertension, and heart disease. Of the 186 prescreened potential participants, 67 were excluded due to related comorbidities. For example, many were excluded due to factors preventing weight-loss (eg, polycystic ovary disease and thyroid disease), heart disease complications, bone and joint problems, and uncontrolled T2D. Of the 77 potential participants who qualified to move on to the in-person SBV, 12 decided not to participate and 8 cancelled or were lost to follow-up. Among those who declined, several cited the barrier of a long commute to the research office. Of all planned recruitment strategies that were implemented, the most time efficient recruitment venues were Filipino community health fairs and cultural events, yielding 32 potential participants. This result may have been due to a combination of factors, including the ease of access to a high density of qualified potential participants in a confined area over a short period of time. In contrast, general public events such as the Daly City Chamber of Commerce events and public dance events that yielded only 6 potential participants, and shopping malls that yielded 17 potential participants, required much more solicitation time on the part of the recruiters compared to other venues and strategies.

Overall, the most effective recruitment strategies were referrals from family, friends, community leaders, and health care providers/clinics, resulting in 53 potential recruits. Filipinos place family and community, along with social support, as a priority in their lives [7]. Thus, the endorsements received from personal and trusted sources may have motivated potential participants to contact our research office.

Flyers posted in hospitals and the health institution’s shuttle buses resulted in 20 potential recruits, whereas posting flyers at community centers and public areas (gyms, coffee shops, and laundromats) were not as effective, yielding only 10 potential recruits. Filipinos with T2D may be more likely to frequent health-related venues compared to more general public areas, thereby explaining the relative differences in the effectiveness between these two recruitment strategies.

Online advertisements through websites, Craigslist, Facebook, and a select mailing service were not as effective as anticipated, compared to in-person recruitment efforts. The social aspect of the personal contact may have encouraged participation versus the relatively impersonal digital media contacts.

### Potential Limitations

In addition to the limitations referenced above, including the study design (eg, unblinded research staff and participants), recruitment issues due to stringent inclusion/exclusion criteria, and adherence to protocol due to lost Fitbit Zips, there are several other potential study limitations. First, it may be difficult for older participants, and those without previous experience using mobile phones and mHealth apps, to comply with this study protocol, thus adversely impacting engagement and retention rates. However, evidence indicates that in a similar mHealth study, older participants and those without previous experience using digital technology were able to adhere to the intervention protocol requirements without influencing outcomes or retention [51]. To further mitigate potential problems using digital technology, family members with digital technology experience were encouraged to partner with participants exhibiting difficulties using the Fitbit Zip or the mHealth app/diary. Second, for those subjects who worked or were unable to attend regular Monday through Friday office hours, weekend and evening in-person office visits were available. Third, the potential subject’s primary care providers were mailed a *Study Provider Letter* to inform them of the subject’s intent to participate. This information offered the primary care provider an opportunity to screen out high risk or inappropriate participants from the study. Additionally, by alerting providers to the nature and overall details of the study, the potential of general practitioners implementing a treatment plan that would bias the study was reduced. Finally, the small sample size and targeted Filipino population living in northern California limits the generalizability of the findings to other populations. However, a major strength of this study is that it has been designed not only to be culturally appropriate for Filipinos, but translatable to other at-risk populations.

### Conclusion

If the PilAm Go4Health Weight Loss Program demonstrates potential efficacy, it would aid in the identification of effective intervention strategies that could significantly reduce risks for metabolic syndrome in Filipinos with T2D. This program will also lay the foundation for a larger full-scale RCT to demonstrate intervention effectiveness, and operational- and cost-effectiveness. Furthermore, if the PilAm Go4Health intervention demonstrates feasibility and acceptability in this high-risk minority population, it could be developed into a sustainable and scalable healthy lifestyle intervention program that could be adapted and widely disseminated to other high-risk racial/ethnic and diverse populations.

## Appendix

Multimedia Appendix 1 PilAm Go4Health Inclusion/Exclusion Criteria.

## Abbreviations

DPP: Diabetes Prevention Program
HbA1c: glycated hemoglobin A1c
mHealth: mobile health
NHLBI: National Heart, Lung, and Blood Institute
PA: physical activity
PilAm Go4Health: Filipino Americans Go4Health Weight Loss Program
RCT: randomized controlled trial
SBV: screening baseline visit
T2D: type 2 diabetes
