# Supplementary material for: Development of a Mobile Phone-Based Weight Loss Lifestyle Intervention for Filipino Americans with Type 2 Diabetes: Protocol and Early Results From the PilAm Go4Health Randomized Controlled Trial
Source: JMIR Res Protoc. 2016 Sep 8;5(3):e178. doi: 10.2196/resprot.5836 (PMC5034152; doi:10.2196/resprot.5836)
Supplement: Multimedia Appendix 1 [file resprot_v5i3e178_app1.pdf]

Appendix A

PilAm Go4Health Inclusion/Exclusion Criteria

Inclusion Criteria: **1)** Self-identified as FA, **2)** Age $\geq$ 18 years, **3)** WHO BMI cut-point for Asians $\geq$ 23 kg/m2 for public health action [20]. 4) Physician diagnosed T2D confirmed by clinical data (e.g. documentation of fasting blood glucose $\geq$100 mg/dL or a positive oral glucose tolerance test (OGTT) $\geq$ 200 mg/dl, HbA1c $\geq$ 6.5%), 5) Treated for T2D with only oral medicine, 6) waist circumference: men > 40 inches, women > 35 inches, 7) Physically inactive per Brief Physical Activity Recall Questionnaire [49], 8) No cognitive impairment per the Mini-Cog test [48]. 9) English speaking, and 10) own a smartphone (iPhone 4s or above, or an android phone), tablet, or computer. Only one member per household is allowed to enroll in the study to avoid cross contamination. *Exclusion criteria:* 1) Uncontrolled T2D (fast blood sugar > 200), 2) Disabilities precluding ability to walk for 20 minutes, 3) Glucose metabolism associated disease (Cushing's syndrome, acromegaly, and pheochromocytoma currently under treatment, chronic pancreatitis), 4) Thyroid disease and polycystic ovary syndrome, 5) Known medical conditions or other physical problems needing a special exercise program (e.g., prior myocardial infarction, history of angioplasty or angina, admission for hospital evaluation of chest pain, use of nitroglycerin for angina, COPD, or uncontrolled hypertension), 6) Recent acute coronary syndrome, revascularization, or congestive heart failure; 7) Participating in a lifestyle modification program, 8) Planning a trip outside the US without access to internet during the 6-month study period, 9) Known eating disorder, 10) Planning a surgery in the next 7-months, 11) Taking long-term antibiotics including HIV-related agents, anti-TB agents (except INH alone as prophylaxis)], or prescription weight-loss medication.
